# Can Exercise Increase Fitness and Reduce Weight in Patients with Schizophrenia and Depression?

**DOI:** 10.3389/fpsyt.2014.00089

**Published:** 2014-07-28

**Authors:** Jesper Krogh, Helene Speyer, Hans Christian Brix Nørgaard, Ane Moltke, Merete Nordentoft

**Affiliations:** ^1^Mental Health Centre Copenhagen, Faculty of Health Sciences, University of Copenhagen, Copenhagen, Denmark; ^2^Department of Endocrinology, Herlev University Hospital, Copenhagen, Denmark; ^3^Research Unit Department P, Aarhus University Hospital, Risskov, Denmark; ^4^Research Center for Health Promotion, University of Roskilde, Roskilde, Denmark

**Keywords:** exercise therapy, schizophrenia, depression, randomised controlled trials, weight loss

## Abstract

**Background:** Psychiatric patients have a reduced life expectancy of 15–20 years compared with the general population. Most years of lost life are due to the excess mortality from somatic diseases. Sedentary lifestyle and medication is partly responsible for the high frequency of metabolic syndrome in this patient group and low levels of physical activity is associated with increased risk of cardiovascular disease, diabetes, and all-cause mortality. This study aimed to review trials allocating patients with either schizophrenia or depression to exercise interventions for effect on cardiovascular fitness, strength, and weight.

**Methods:** We searched PubMed, Embase, and PsycINFO including randomized clinical trial allocating patients with either schizophrenia or depression to isolated exercise interventions.

**Results:** We identified five trials including patients with schizophrenia (*n* = 94) and found little evidence that exercise could increase cardiovascular fitness or decrease weight. Nine exercise trials for patients with depression (*n* = 892) were identified increasing cardiovascular fitness by 11–30% and strength by 33–37%. No evidence in favor of exercise for weight reduction was found.

**Conclusion:** Based on the current evidence isolated exercise interventions are unlikely to improve cardiovascular fitness or induce weight loss in patients with schizophrenia. In patients with depression, exercise interventions are likely to induce clinically relevant short term effects, however, due to lack of reporting, little is known about the effect on weight reduction and cardiovascular fitness. Future exercise trials regarding patients with mental illness should preferably measure changes in cardiovascular strength, repetition maximum, and anthropometric outcomes. Ideally, participants should be assessed beyond the intervention to identify long lasting effects.

## Introduction

There is a known increased morbidity and mortality due to illnesses among people with mental disorders. A large Nordic study has recently shown that psychiatric patients have a reduced life expectancy of 15–20 years compared to the general population (1). This excess mortality is to some extent explained by suicide and accidents, but most of lost year of life is due to excess mortality from somatic disease (2). Detailed analysis of mortality from specific causes of death shows that this excess mortality is caused by virtually any physical illness (3).

A study of patients treated with antipsychotic medication in outpatients’ services in Copenhagen has shown that the prevalence of metabolic syndrome is twice as high in these patients compared with the general population (4). The metabolic syndrome is a cluster of symptoms related to increased risk of cardiovascular events, type II diabetes, and all-cause mortality. The definition of metabolic syndrome varies, but most includes central obesity, raised blood pressure, high fasting glucose levels, and dyslipidemia (raised triglycerides and lowered high-density lipoproteins) (5). The high prevalence of metabolic syndrome among mentally ill is presumably partly due to weight gain and impaired glucose tolerance, which can be side effects of antipsychotic medication (6). Another reason for the high morbidity and mortality may be the lacking treatment of physical illnesses (7). However, there is also reason to believe that lifestyle factors such as smoking, diet, and exercise habits play a role in the excess mortality, morbidity, and high rates of illnesses. Sedentary lifestyle and poor diet is also more frequent than in the general population (8, 9). Low levels of physical activity are associated with increased risk of cardiovascular disease, diabetes, and all-cause mortality (10, 11). However, adherence to behavioral interventions in both the general population as well as in patients with mental illness is a major issue (12). The excess morbidity in patients with severe mental illness can be attributed to unhealthy lifestyle, medication, and that patients with mental illness do not receive same treatment for physical illnesses as mentally healthy patients. In theory, these are all modifiable factors.

With our current knowledge, there is no reason to believe that the physiological benefit of exercise should be different in mentally ill compared to healthy subjects. However, psychiatric symptoms (e.g., anxiety and lack of energy), medication adverse events (e.g., weight gain and sedation), and factors such as social isolation and difficulties in planning, are issues for many patients with either depression or schizophrenia. Due to these challenges, results from lifestyle interventions in the mentally healthy populations cannot be extrapolated to mentally ill populations.

Exercise, which has a low number of side effects, is affordable for most health care systems and is increasingly an objective for research in this patient group. Furthermore, isolated exercise interventions are simple to implement, less costly, and demands less education of staff compared to more complex interventions. The aim of this study was to review the literature on the effect of exercise interventions on fitness variables such as maximal oxygen uptake and strength. Secondly the aim was to assess the effect of exercise interventions on weight and body mass index (BMI). Also, adherence to exercise interventions will be reviewed.

## Methods

This study was as a systematic review for the primary aims. We included randomized clinical trials allocating patients diagnosed with schizophrenia, bipolar disorder, or depression according to a diagnostic system (i.e., DSM or ICD) to an exercise intervention or a control group/treatment as usual or to an exercise intervention as add-on treatment. The trials had to report outcomes on change in maximal oxygen uptake, repetition maximum or similar in order to be included. We also searched references in relevant papers for additional trials. Secondly, we included studies aiming to identify exercise participation.

### Search strategy

We searched PubMed, Embase, and PsycINFO using the following text word terms: (mental disorders) AND (health promotion OR exercise physical OR physical therapy OR physical activity OR sports OR “life style intervention” OR life style OR food intake OR diet OR dietary OR nutrition OR nutritive OR nutrition therapy OR cognitive therapy OR behavioral therapy) AND (bipolar disorder OR schizophrenia OR psychoses OR depression OR depressive disorder OR “severe mental disorder*”) AND (physical fitness OR physical health OR metabolic syndrome OR cardiometabolic OR cardiovascular OR BMI OR body mass index OR body fat OR weight loss OR weight maintenance OR waist to hip ratio OR “study outcome” OR “study intervention”). The main search was conducted in the summer of 2012 and a follow-up search was conducted in March 2014 in PubMed.

### Data extraction

Data of participants’ baseline characteristics, type of intervention, attendance, and effect on aerobic fitness or strength, and metabolic variables. We expected high heterogeneity of trial interventions, in reporting of effects, and chosen effect estimates and *a priori* abstained from performing a meta-analysis.

## Results

The updated search in March 2014 resulted in 5742 hits. Five (13–17) trials reporting the effect of exercise interventions for schizophrenia fulfilled the inclusion criteria and were included. Nine trials (18–26) reporting the effect of exercise in patients with depression fulfilled the inclusion criteria and were included. No trials including patients with bipolar disorder fulfilled the inclusion criteria.

### The effect of exercise on fitness or strength in patients with schizophrenia

Five trials allocating a total of 141 patients to an exercise intervention or a control group reported the effect on fitness. A summary of these trials can be seen in Table 1. Four studies used aerobic exercise (15–17) and one was a mixed intervention of both strength training as well as aerobic training (14). The vast majority of included participants were outpatients with a mean age between 29 and 52 years. Of the five trials, a recent Dutch study (13) is the most interesting due to its size. In that study, 63 patients were randomly assigned to a training (*n* = 31) or occupational therapy (*n* = 32). The training program consisted mainly of exercises that were intended to increase cardiovascular fitness. There was supervised training twice a week for 6 months. After 6 months, the fitness in the exercise group was significantly higher compared with the control group. This difference was not due to an increase in fitness as a result of training, but a decrease in fitness observed in the control group. However, participants who had an attendance of <50% were excluded from this calculation. The authors conclude that the intervention helped maintain an otherwise declining fitness. The remaining four trials included between 12 and 30 participants. None of these trials found a significant effect of exercise on fitness or strength. It should be noted, however, that all four trials reported a non-significant increase in aerobic fitness.

**Table 1:** **Randomized clinical trials investigating the effect of exercise interventions in patients with schizophrenia**.

Trial	Participants	Number participants randomized	Number of participants in included in final analysis	Intervention	Attendance	Results fitness^a^	Results weight^a^
Scheewe et al. (13)	Outpatients	E = 31	E = 17	Supervised aerobic exercise. Two sessions/week for 6 months	E: 20/31 participants attended >50%. Mean attendance was for these 20 participants was 1.5 session/week	Change in aerobic fitness was significantly higher in E-group	No significant effect on BMI or waist circumference
	Mean age: 29.7	C = 32	C = 16				
	Female: 17/63 (27%)						
Battaglia et al. (15)	Outpatients	E = 12	E = 10	Supervised soccer training. Two sessions/week for 3 months	Exercise group: 83% participated in >80% of sessions	No significant effect on 30 m sprint performance	Significant reduction in weight and BMI of 4.6% E-group
	Mean age: 35.5	C = 11	C = 8				
	Female: 0/23 (0.0%)						
Marzolini et al. (14)	Outpatients	E = 7	E = 7	Supervised mixed aerobic and strength training. Two sessions/week for 3 months	Exercise group: mean attendance was 1.44 sessions/week	E-group increased 6 min walking test by 28 m and a 28% increase in strength	No significant effect on weight or waist circumference
	Mean age: 44.9	C = 6	C = 6				
	Female: 7/15 (47%)						
Beebe et al. (17)	Outpatients	E = 6	E = 4	Supervised aerobic exercise. Three sessions/week for 4 months	Not reported	E-group could walk 29 m longer in a 6-min test. Not significant	No significant effect on BMI. A significant decrease in body fat of 3.7% in E-group
	Mean age^b^: 52	C = 6	C = 6				
	Female^b^: 2/10 (20%)						
Skrinar et al. (16)	Outpatients and in-patients	E = 15	E = 9	Supervised aerobic exercise. Four sessions/week for 3 months	Exercise group: mean attendance was 2.6 sessions/week	E-group increased exercise performance from 153 to 161 W on bicycle test. Not significant	No significant effect on weight, BMI, or % body fat
	Mean age: 38	C = 15	C = 11				
	Female: 20/30 (66.7%)						

*E, exercise group; C, control group*.

*^a^Significant’ refers to between-group differences*.

*^b^Age and gender is only reported for completers*.

### The effect of exercise on body weight in patients with schizophrenia

As illustrated in Table 1, four of five trials did not find that exercise reduced weight or BMI. However, one study with soccer intervention found a significant reduction of 4.6% in weight and BMI (15) and another trial found a 3.7% decrease in body fat (17).

### Participation in exercise interventions in patients with schizophrenia

Four of five trials report on attendance. In the Dutch study (13), only participants attending more than 50% of sessions are included in the assessment. In this “completers” group, the average attendance is 1.5 session/week. In Battaglia et al. (15) using soccer, training 83% participated in more than 80% of the 24 offered sessions. In the remaining trials, the mean participation ranged between 1.4 and 2.6 session/week. In the trial by Marzolini et al. (14), they registered barriers for two attendances based on participants’ feed-back and found the top four reasons to be unknown (47%), medical issues including hospitalization (27%), supervised trip or family visit (8%), and medical appointment (4%).

### The effect of exercise on fitness or strength in patients with depression

As reported in Table 2, nine trials reporting on the effect of exercise on fitness or strength was identified. These 9 trials included a total of 950 patients. Six trials used aerobic interventions (19–22) and two trials investigated the effect of strength training (18, 23). In one trial (27), the participants were encouraged to engage in preferred exercise activities offered in the community. The range of mean age was between 36 and 71 years of age. Two of nine trials investigated the effect of exercise in inpatients (24, 26).

**Table 2:** **Randomized clinical trials reporting effect of exercise interventions on physical fitness or strength in patients with depression**.

Trial	Participants	Number of participants randomized	Number of participants included in final analysis	Intervention	Attendance	Results fitness	Results weight
Krogh et al. (19)	Outpatients	E = 56	E = 56	Supervised aerobic exercise. Three sessions/week for 3 months	Average number of attended sessions 1.1/week	Significant increase in maximal oxygen uptake by 13% in E-group compared to C-group	No effect on weight or BMI. Significant reduction in waist circumference – 2 cm
	Mean age: 41.6	C = 59	C = 59				
	Female: 77/115 (70%)						
Chalder et al. (25, 27)	Outpatients	E = 182	E = 151	Exercise coaching face to face and by telephone during an 8-months period	Not reported	Significant effect of intervention: in E-group 63% of participants were physically active >150 min/week compared to 49% in C-Group	Not reported
	Mean age: 39.9	C = 179	C = 165				
	Female: 122/361 (33.8%)						
Krogh et al. (20)	Outpatients	E = 55	E = 55	Supervised aerobic exercise. Two sessions/week for 4 months	Average number of attended sessions was 1.0/week	Significant increase in maximal oxygen uptake by 11% in E-group compared to C-group	Not reported
	Mean age: 36.7	C = 55	C = 55				
	Female: 77/110 (70%)						
Knubben et al. (24)	Inpatients	E = 20	E = 20	Daily supervised aerobic exercise sessions for 10 days	Not reported	No difference in maximal oxygen uptake	Not reported
	Mean age: 49.5	C = 18	C = 18				
	Female: 21/38 (55.3%)						
Blumenthal et al. (22)	Outpatients	E = 51	E = 51	Supervised aerobic exercise. Three sessions/week for 4 months	Median number of attended sessions: 2.3/week	Significant increase in maximal oxygen uptake in E-group compared to C-group 9.1%	Not reported
	Mean age: 52.0	C = 49	C = 49				
	Female: 23/100 (23%)						
Singh et al. (23)	Outpatients	E = 20	E = 18	Supervised strength training. Three sessions/week for 2 months	Median number of attended sessions: 3.0/week	Significant increase in muscle strength by 37% in E-group	Not reported
	Mean age: 69.0	C = 20	C = 19				
	Female: 21/40 (0.53%)						
Blumenthal et al. (21)	Outpatients	E = 55	E = 55	Supervised aerobic exercise. Three sessions/week for 4 months	Average number of attended sessions: 2.7/week	Significant increase of maximal oxygen uptake by 11% in E-group	Not reported
	Mean age: 57.0	C = 48	C = 48				
	Female: 74/103 (71.8%)						
Singh et al. (18)	Outpatients	E = 17	E = 15	Supervised strength training. Three sessions/week for 2.5 months	Median number of attended sessions: 3.0/week	Significant increase in muscle strength by 33% in E-group	Not reported
	Mean age: 71.3	C = 17	C = 15				
	Female: 20/32 (71.8)						
Martinsen et al. (26)	Inpatients	E = 28	E = 24	Supervised aerobic exercise. Three sessions/week for 9 weeks	Not reported	Significant increase in maximal oxygen uptake by 30% in the E-group	Not reported
	Mean age: 40.0	C = 21	C = 19				
	Female: not reported						

*E, exercise group; C, control group*.

*Authors estimate based on graph in original report*.

Five of six trials offering aerobic exercise found a positive and significant effect on fitness levels in the range of 11–30% in maximal oxygen uptake. The two trials investigating the effect of strength training both found an increase in strength between 33 and 37%.

The largest study to date is the British TREAD study (25) using motion advisors trying to facilitate more physical activity in daily life in patients. Patients could choose how they wanted to increase their physical activity, and were supported in their attempt to this lifestyle change through meetings or telephone conversations with their exercise counselor. In Great Britain, authorities recommend that adults are active approximately 150 min/week, and the TREAD study could, on the basis of questionnaires, assess the number of participants, respectively exercise and the control group that met these requirements after intervention. It turned out that the chance of a participant in the exercise group met the official recommendation for physical activity after the intervention was twice higher (OR 2.27, 95% CI 1.32–3.89, *p* = 0.003) compared to a participant from the control group.

### The effect of exercise on body weight in patients with depression

Only one trial reports the effect of exercise on body weight (19) and it found no effect on weight or BMI. However, the intervention group reduced their waist circumference with 2 cm post-intervention.

### Participation in exercise trials with depressed patients

Six trials report on adherence (18–23). Mean attendance in aerobic trials ranged from 1.1 to 2.7 sessions/week. The two strength training trials reported a median attendance of 3 sessions/week. None of the trials report on reasons for lack of attendance.

## Discussion

Trials examining the effect of exercise in patients with schizophrenia are small in number and size. There is little evidence that exercise interventions will increase cardiovascular fitness or lead to decreased weight in this patient group. There is convincing evidence that depressed outpatients participating in strength or aerobic training will improve their fitness in terms of maximal oxygen uptake or repetition maximum. However, there is little evidence that exercise interventions in patients will depression will decrease their weight.

Four of five trials assessing exercise in patients with schizophrenia found a positive effect on fitness levels and lack of significance is likely to be a type II error due to the small sample sizes. However, in the larger study by Scheewe et al. (13), the significant differences in aerobic fitness were due to a decrease in fitness in the control group and not an increase in the intervention group. In six trials including patients with depression to aerobic exercise, five had a significant increase maximal oxygen uptake. The trials that did not improve aerobic fitness was a 10-day intervention of inpatients by Knubben et al. (24), which is probably too short an intervention to gain physiological improvement. In a Finnish study of 1639 men (42–60 years) without a history of type II diabetes or atherosclerotic cardiovascular maximal oxygen uptake was associated with 16% reduction of all-cause mortality per one increment in MET (3.5 ml oxygen/min/kg) during an average follow-up of 16 years (28). In the DEMO-I trial, the aerobic exercise groups increased their aerobic capacity equivalent to an increase of 0.83 METs and in the DEMO-II trial, the increase was equivalent to 0.97 METs (19, 20). This suggests that the increase in cardiovascular fitness observed in exercise trials of depressed patients is clinically relevant. On the other hand, no data exists on maximal oxygen uptake beyond the intervention and therefore the current evidence does not allow conclusions or speculations whether the observed increases in cardiovascular fitness is transformed to any benefit for the patients on hard endpoints.

Patients with schizophrenia are unlikely to decrease weight in response to exercise interventions. For patients with depression only one trial reported on weight and found no weight reducing effect of exercise participation (19). However, in that trial a reduction of waist circumference was found in the intervention group reflecting a potential redistribution of weight from abdominal fat to muscle. A recent meta-analysis confirms the effect of both moderate to high intensity aerobic training and strength training for reduction of visceral fat (29). Isolated aerobic exercise as an intervention for weight loss in overweight or obese populations has in meta-analysis been found to reduce weight by 1.6 kg, waist circumference of 2.1 cm, diastolic blood pressure with 1.8 mmHg, and total cholesterol with 1.54 mg/dl for 6 month programs (30). These results suggest that even in mentally healthy populations an isolated aerobic intervention is not likely to induce dramatic changes in weight or waist circumference.

These findings open a discussion whether multiple interventions should be employed, i.e., combined exercise and diet interventions. In mentally healthy populations, the combination of exercise and diet interventions for weight loss has been shown to be superior to exercise alone (31). A trial of patients with severe mental illness allocated to an intervention containing both of advice on diet as well as exercise obtained a weight loss of 3.2 kg after 18 months duration (32). A meta-analysis of 17 non-pharmacological interventions in patients with severe mental illness supports these findings with an overall weight reduction of 3.12 kg (33). However, only five trials reported the effect beyond the intervention with conflicting results on weight and BMI (33). For schizophrenia, only a few and mainly very small trials fulfilled the inclusion criteria for this review. The main reasons for exclusion of trials were lack of diagnosis (e.g., not specifying diagnostic instrument) and the use of mixed interventions (e.g., exercise and diet versus treatment as usual). The results from this review should be interpreted in that context. Taken together, isolated exercise interventions in patients with schizophrenia or depression are unlikely to induce clinically relevant weight loss. On the other hand, there is evidence in favor of mixed interventions for weight loss.

Regarding adherence to exercise intervention in patients with schizophrenia, there was a trend for high attendance in small trials whereas larger trials had lower attendance. Previous studies have tried to identify barriers to physical activity and limited experience of physical, activity engagement, impact of the illness and effects of medication, and anxiety is mentioned by one study (34) and partially confirmed in a smaller study (35). The authors have recently investigated predictors of adherence in exercise trials including patients with depression and found that age and satisfaction with the intervention was associated with higher adherence (36). The age aspect could explain some of the variation observed in attendance in trials with depressed patients. Unfortunately, the majority of included trials do not mention considerations regarding attendance.

Based on the current evidence isolated exercise interventions are unlikely to improve cardiovascular fitness or induce weight loss in patients with schizophrenia, but there are few studies and thus a rather small evidence base. In patients with depression, exercise interventions are likely to induce clinically relevant short term effects, however, due to lack of reporting, little is known about the weight reduction. Future exercise trials in patients with mental illness should preferably measure changes in cardiovascular strength, repetition maximum, and anthropometric outcomes. Ideally, participants should be assessed beyond the intervention to obtain knowledge on lasting effects.

## Conflict of Interest Statement

The authors declare that the research was conducted in the absence of any commercial or financial relationships that could be construed as a potential conflict of interest.
